# Psychometric properties of the Alcohol Use Disorders Identification Test (AUDIT) and prevalence of alcohol use among Iranian psychiatric outpatients

**DOI:** 10.1186/s13011-018-0141-x

**Published:** 2018-01-30

**Authors:** Simasadat Noorbakhsh, Jamal Shams, Mohamadmahdi Faghihimohamadi, Hanieh Zahiroddin, Mats Hallgren, Hakan Kallmen

**Affiliations:** 1 Behavioral Sciences Research Center of Shahid Beheshti University of Medical Sciences, Tehran, Iran; 2 Department of Public Health Sciences, Karolinska Institutet, Stockholm, Sweden; 3 Department of Clinical Neuroscience, STAD, Centre for Psychiatry Research, Karolinska Institutet & Stockholm Health Care Services, Stockholm County Council, Stockholm, Sweden

**Keywords:** Alcohol Use Disorders Identification Test (AUDIT), Alcohol use disorder, Prevalence, Psychometric properties

## Abstract

**Background:**

Iran is a developing and Islamic country where the consumption of alcoholic beverages is banned. However, psychiatric disorders and alcohol use disorders are often co-occurring. We used the Alcohol Use Disorders Identification Test (AUDIT) to estimate the prevalence of alcohol use and examined the psychometric properties of the test among psychiatric outpatients in Teheran, Iran.

**Methods:**

AUDIT was completed by 846 consecutive (sequential) patients. Descriptive statistics, internal consistency (Cronbach alpha), confirmatory and exploratory factor analyses were used to analyze the prevalence of alcohol use, reliability and construct validity.

**Results:**

12% of men and 1% of women were hazardous alcohol consumers. Internal reliability of the Iranian version of AUDIT was excellent. Confirmatory factor analyses showed that the construct validity and the fit of previous factor structures (1, 2 and 3 factors) to data were not good and seemingly contradicted results from the explorative principal axis factoring, which showed that a 1-factor solution explained 77% of the co-variances.

**Conclusions:**

We could not reproduce the suggested factor structure of AUDIT, probably due to the skewed distribution of alcohol consumption. Only 19% of men and 3% of women scored above 0 on AUDIT. This could be explained by the fact that alcohol is illegal in Iran. In conclusion the AUDIT exhibited good internal reliability when used as a single scale. The prevalence estimates according to AUDIT were somewhat higher among psychiatric patients compared to what was reported by WHO regarding the general population.

## Background

In many developed countries, Alcohol Use Disorder (AUD) is one of the most prevalent psychiatric disorders and is associated with considerable disability [1]. It also causes a heavy financial burden on health systems [2]. In DSM-IV-TR, AUD included two types of alcohol related diagnoses, alcohol dependence and alcohol abuse, with special criteria for each [3]. In the DSM-5, this concept was merged into a single disorder named AUD, with sub-classifications of mild (the presence of 2–3 symptoms), moderate (4–5 symptoms), and severe (6 symptoms or more) [4]. In developing countries, the AUD prevalence rate is often lower than in developed ones [5], yet it remains problematic. After opioids, AUD is the most serious addictive problem in Iran [6]; a developing Islamic country located in the Middle East where the majority of Iranians are Muslim. According to Islamic law, the use of alcoholic beverages is forbidden; moreover selling, buying or manufacturing alcoholic beverages is also illegal [7]. Thus, alcohol is not readily accessible and there are no advertisements (TV, magazines, etc.) or promotional sales. However, illegal alcoholic beverages are available in Iran and include: neutral spirits (purchased from drug stores), Aragh Sagi (handmade, obtained from distillation of raisins containing at least 65% pure ethanol), wine (handmade and bottled), whiskey (an illegal importation, mainly from Iraq), beer (handmade and canned), etc. Normally, the price of these products is beyond the purchasing power of most people. The Iranian population consist of more than 90% lifetime abstainers yet the average alcohol consumption was about 1 l per capita, but among alcohol consumers the average consumption was 25 l per capita [8], indicating that alcohol consumption is a problem in selected groups. Addiction problems in Iran consist mainly of illicit drug use. Research activity and the dependency health care system are also focused on problems associated with illicit drug use. The only reliable report on health and alcohol is the Global Status Report prepared by the World Health Organization [5]. Studies on sub populations such as high-school or college students generally placed alcohol use/abuse after cigarette and hookah -a stemmed instrument for smoking tobacco whose vapor or smoke is passed through a water glass basin before inhalation- smoking [9–11] and have estimated the lifetime prevalence of alcohol use to be between 17 and 25% [9, 12]. According to latest global report of alcohol consumption by the WHO [5], in 2010, 91% of men and 95% of women (both aged 15 years or more) residing in Iran were lifetime abstainers, and the prevalence rate of AUD was less than 0.5%. Currently, there is no national system for monitoring alcohol consumption or support for community prevention [5]. However, in 2011 the Iranian household Mental Health survey was conducted. It showed that the 12 month prevalence of alcohol consumption in the Iranian population aged between 15 and 64 years was 5.7% and that the prevalence of AUD, according to DSM-5, was 1.3% [13]. Authors showed that individuals who were young, unmarried, male, and whose with mental health problems had a higher risk for AUD. Nikifarjam et al., (2017) estimated 12-month prevalence by using a network scale up method, where participants were asked how many people in their social network consumed alcohol. The 12 month prevalence of alcohol consumption in the Iranian population was estimated to be 2.31%, but in young men aged 18–30 years it was estimated at 7% [14].

Comorbid psychiatric disorders and AUD are highly prevalent. In the United Kingdom, it was estimated that 30% of alcohol dependent individuals had a co-occurring psychiatric disorder. In Australia, the corresponding prevalence is estimated to be 12–25% [15], and the lifetime prevalence of alcohol consumption is higher in psychiatric patients than in the general population [16]. This comorbidity causes additional financial burdens [17]. Due to the high comorbidity of AUDs with other psychiatric disorders, early detection of alcohol consumption can be helpful in increasing the quality of treatment through collaboration with other specialists [18] and more specialized pharmacotherapy, and by reducing the barriers to treatment services, and informing patients about drug interaction.

The ban on alcohol consumption and the high proportion of co-occurring psychiatric disorders and AUD makes it possible that the occurrence of alcohol consumption is higher among those who suffer from psychiatric disorders and they are also often socially marginalized. According to Morisano [18] patients with this comorbidity need a particular treatment why a valid and reliable assessment of alcohol consumption is necessary.

Although screening and brief interventions (SBI) with motivational interviews are often effective in helping patients to reduce or stop drinking [19], identifying problems related to alcohol consumption is often difficult [20]. Moreover, SBI has been shown to be difficult to implement in routine health care. A more reliable method is electronic SBI [21] which is shown to help reduce alcohol consumption. Various screening instruments have been developed to detect alcohol use, but most have limitations. For instance, the Michigan Alcoholism Screening Test (MAST) [22] is long and time-consuming (24 questions) and difficult to conduct in outpatient settings which are often crowded and time-limited. Similarly, the CAGE/T-AGE [23] is short but cannot identify differences between alcohol dependence and alcohol abuse. However, the Alcohol Use Disorders Identification Test (AUDIT) does not have these limitations and can be used to identify AUD according to DSM-5 [24]. AUDIT was specifically developed on request by the WHO [25] and assess life-time alcohol consumption and alcohol related harms during the past year. The 10-item test was developed in six different countries and cultural groups to be a valid assessment instrument and a review verifies its validity and reliability in different populations [26]. AUDIT is considered a suitable instrument within treatment settings and for patients with psychiatric disorders [27] It is a psychometrically sensitive and specific test with a cut-off of 8 points for hazardous drinking [28–30] and 20 for alcohol dependence [23]. In a sample of 250 patients at a treatment clinic in Teheran and diagnosed as alcohol dependent, the average AUDIT score was above 28 [31]. Although underreporting of alcohol consumption among patients in treatment can be expected, especially if alcohol consumption is banned - it is reported that self-report tools have significant advantages such as higher sensitivity than observational and laboratory data [32]; they are also cheaper, faster, and simple to administer.

Both the validity and reliability of AUDIT have been studied in many countries and cultures [26], and in medical settings, however there is currently no available data about alcohol consumption among Iranian psychiatric patients. Thus, the current study aimed to investigate the psychometric properties of AUDIT in psychiatric outpatients in Tehran (Iran) and to estimate the prevalence of AUD in this cohort.

## Methods

### Administration and participants

The present study was part of a pilot study which aimed to prepare an instruction to detect AUDs for psychiatric settings in Iran. Due to the limitations confronting this research (e.g. patients’ fear and conservatism in disclosing the performance of a non-religious or illegal action), psychiatrist interviews were considered the optimal data collection method. AUDIT was presented as an oral interview to patients referred to the general psychiatry clinic of Imam Hossein Hospital from May to November 2016. This is a training hospital under the supervision of Shahid Beheshti University of Medical Sciences located in eastern Tehran which covers patients from central, north-eastern, south-eastern and eastern Tehran. All patients referred for the first time or for follow-up were invited to participate in the research. Participants were consecutive (sequential) patients. Only those who agreed to fully participate were considered. Since participants were from a general psychiatry clinic with all occurring diagnoses they can be considered a representative sample of Iranian psychiatry out-patients in Teheran. If they did not respond to an item they were reminded to do so In order to calculate the test-retest reliability, we took advantage of the questionnaires from 19 participants which had a gap of more than 14 days between scheduled appointments, We also conducted the test-retest on 11 participants from an another treatment center (Andishe-No). Most of the data were collected from an addiction treatment center for patients who used opioids and narcotics. They usually received methadone as a maintenance therapy, so the center was not for all types of mental disorders. We proposed the brief interventions for alcohol ab/use after conducting retest. In total 30 patients participated in the test-retest analysis.

Inclusion criteria were age 18 years and over, residency in Iran, and full understanding of the Persian language. Exclusion criteria were cognitive problems interfering with participants understanding of the questions and any condition which could lead to inaccurate responses, as assessed by a psychiatrist. The ethics committee of Shahid Beheshti University of Medical Sciences approved the research proposal (No. 1379). All participants provided written informed consent. If the participants were at risk for AUD, brief interventions or specialist referral were considered.

We sampled patients referred to the outpatient clinic for the purpose of seeing a psychiatrist. When the psychiatrist finished the session, s/he gave a brief explanation about the study and asked the AUDIT questions. In total, 48 patients were excluded at this stage because; 1) they declined to participate in the study, and 2) they offered inconsistent and/or unreliable responses about their alcohol and drug use (according to the psychiatrist) compared with reports from their family and important others. There were 846 consecutive patients included in the sample, 354 men and 490 women, and 2 who did not disclose their gender.

The sample size was sufficient for the statistical analysis which includes confirmatory and exploratory factor analyses of AUDIT. In determining the psychometric characteristics, an item to person ratio of 10:20 is recommended [33]. Taking into account the following parameters – SD 0.5, error margin + 5%, and statistical significance 0.05 - we determined that a minimum sample size of 384 would be acceptable for these analyses. However, we increased this to 846 to increase accuracy of the estimates.

#### Measures

##### Demographic and psychiatric diagnosis

During an interview, a psychiatrist’s assistant collected information on the patients’ age, gender, marital status and educational level. A psychiatrist determined the patient’s diagnosis based on DSM-5 criteria. These data were compared and cross-checked with patient’s files.

##### Alcohol use disorders identification test (AUDIT)

AUDIT is a 10 item scale consisting of 3 dimensions; items 1–3 assess alcohol consumption, items 4–6 assess alcohol dependence, and items 7–10 assess the presence of alcohol-related problems. Questions 1–8 are scored on a 5-point scale ranging from 0 to 4, and questions 9 and 10 are scored 0, 2 and 4 respectively. As a result, 40 is the highest score that can be obtained from AUDIT [34]. AUDIT questions and their Persian translation are presented in Table 1. The questionnaire correlates highly with other alcohol screening tools [35]. Scores above 8 indicate a high risk of AUD in psychiatric patients [28–30]. A high internal consistency (0.75 to 0.94) has been reported in various studies [28, 35, 36]. Three types of factor solutions(1, 2 and 3 factors, respectively) have been indicated in previous factor analytic studies [37].

**Table 1:** Descriptives for demographic variables

	N	Age (SD)	Years of education (SD)	Marital status
Men	354	37.31 (12.99)	9.92 (5.13)	Single 49.6% (11.717) Married 48.7% (115) Other 1.7% (4)
Women	490	41.26 (14.13)	9.45 (5.54)	Single 26.7% (87) Married 61.7% (201) Other 11.7% (39)

The table shows number of participants, mean age (SD), mean years of education (SD), and marital status of men and women

##### Translation into Persian

Two English language experts translated the English version of AUDIT into Persian. Then, two bilingual scholars back-translated the Persian version to English separately. Finally, a PhD psychologist and a psychiatrist with a good command of English and with expertise in substance abuse identified and moderated any discrepancies and finalized the Persian version. During the translation process, questions 1, 2 and 3 were modified in order to fit the Iranian culture criteria. Specifically in question 2 and 3 the standard units of alcohol use were modified according to be consistent with the availability of Iranian alcoholic drinks. Both versions are presented in Table 2. The standard units of alcohol vary between countries but are mainly between 10 and 12 g of 100% ethanol per drink. The most common beverages, Aragh Sagi and neutral spirits, contain at least 65% pure ethanol.

**Table 2:**
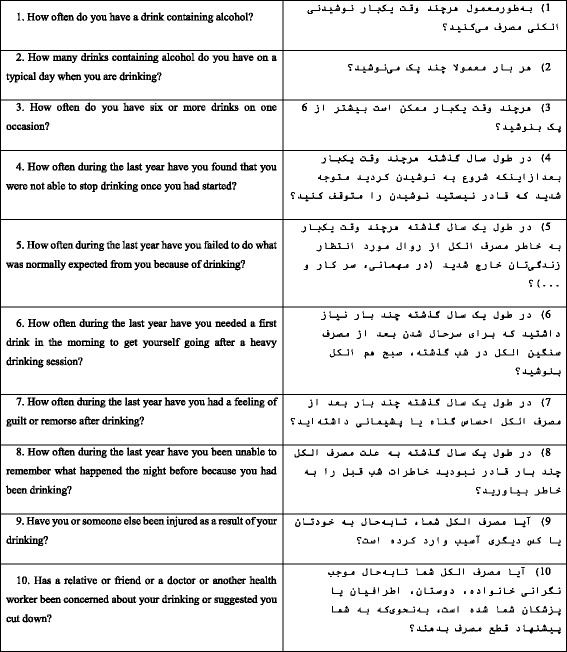
The AUDIT questionnaire items in original (English) and translated to Persian

##### Data analysis

To evaluate the psychometric properties of AUDIT, we computed internal consistency, Confirmatory and Exploratory factor analyses.

Confirmatory Factor Analyses were calculated using STATA version 13 and all other analyses were calculated in SPSS version 22.0. Descriptive statistics (means, frequencies, and SDs) are used to present the demographic and AUDIT data. With non-normally distributed data, Mann-Whitney U-test was applied to test gender difference in AUDIT scores. Independent sample t-tests assessed differences in age and education and the equality of distribution of marital status between men and women was tested by using Chi-square test.

Cronbach’s α was used to calculate AUDIT internal consistency. Scores range between 0 and 1, with 0.6 to 0.8 indicating acceptable reliability. A score of 0.8 or higher indicates good reliability. A score of 0.95 or higher likely indicates that the items are redundant.

As noted, three models have been suggested. The first model, suggested by Saunders et al. [25] consists of three factors: alcohol consumption (questions 1–3), drinking behavior/dependence (questions 4–7), and alcohol-related problems or consequences (questions 8–10). The second model (2 factors) includes alcohol consumption (questions 1–3) and alcohol related problems (questions 4–10) [26, 38]. The third model includes all questions (1–10) loading to a single factor [30, 39]. Confirmatory Factor Analysis was performed to test the fit of these theoretical models to the data and to assess the construct validity of the questionnaire. To explore the factor structure of AUDIT in this sample we calculated an exploratory Principal Axis Factoring (PAF) with promax rotation.

To test the stability of AUDIT over time, test-retest reliability was examined by calculating Cohens Kappa which generates a number between -1and 1, with 1 being a perfect concordance between the test and the retest [40].

Risk for alcohol related harms is generally recognized with scores of 8 points or more for men, and 6 or more for women [41].

## Results

Table 1 shows participant sociodemographic data. A significant difference in the distribution of marital status was observed (χ2 = 45.00, df = 2, *p* < 0.001). More women than men were married or in another relationship. Women were significantly older than men (*t* = 4.192, df = 794, *p* < 0.001) but there was no difference in length of education (*t* = 1.24, df = 761, *p* = 0.22).

AUDIT scores are shown in Table 3. Data indicates a high skewness in the distribution of scores. The proportion of alcohol consumers among psychiatric outpatients was 19% among men and 3% among women. In the total sample, the proportion of hazardous consumers was about 12% among men and about 1% among women. Among those who reported drinking any alcohol, 60% of men and 30% of women were risk consumers. This might explain the skewed distribution in these data. Earlier AUDIT studies have mainly reported means and SDs. Due to the non-normal distribution, we also report the median and inter-quartile range. The more stable estimates denotes by the 50th and the range between 25th and 75th percentiles of the distribution of AUDIT scores show that both are 0. Mann-Whitney U-test showed a significant difference in ranks of AUDIT scores between genders (*p* < 0.001).

**Table 3:** AUDIT scores for men and women

Gender	Proportion score > 0	Proportion hazardous consumers	Median	IR	Mean	SD
Men	19.0	11.6	0.0	0.0	2.8	7.8
Women	3.1	0.6	0.0	0.0	0.1	0.9

The table shows proportion (%) scored > 0, Proportion (%) Hazardous consumers > 8 points for men and > 6 for women, median and Inter-quartile range (IR), Mean and standard deviation (SD)

A test of the construct validity of the Persian version of AUDIT was made using a confirmatory factor analysis by assessing the fit of one, two and three factor solutions (Table 4).

**Table 4:** Confirmatory Factor Analysis (CFA)

	Chi-square	df	*p*	RMSEA	CFI	TLI
1 factor	2303	35	< 0.001	0.28 (.268–.287)	0.80	0.75
2 factors	1441	34	< 0.001	0.22 (.212–.232)	0.84	0.88
3 factors	1364	32	< 0.001	0.22 (.212–.232)	0.88	0.84

Table shows the fit of the one, two and three factor structure of AUDIT. Chi-square, degrees of freedom (df), *p*-value, Root Mean Squared Error of Approximation (RMSEA), Comparative Fit Index (CFI) and Tucker-Lewis Index (TLI)

All three factor structures of AUDIT mentioned in earlier studies [35] were significantly different from the data and showed a poor fit to the data. However, an explorative principal axis analysis showed a one-factor solution explaining 77.7% of the covariance (Table 5). This result seems to be contradictory but may be due to inter-item correlations that were not permitted in the CFA model.

**Table 5:** A single principal axis factor solution of AUDIT

AUDIT questions	Factor loadings In a single factor	Item-total correlation
Item 1	.853	.831
Item 2	.830	.852
Item 3	.899	.899
Item 4	.943	.916
Item 5	.925	.897
Item 6	.856	.821
Item 7	.831	.804
Item 8	.845	.813
Item 9	.809	.804
Item 10	.880	.872

The internal consistency (Cronbach’s alpha) was excellent (0.96). We also calculated Cohens Kappa to obtain test-retest reliability on a particular group. The stability over time was substantial, K = 0.64 [40].

## Discussion

Previous studies have shown that the co-occurring use of alcohol and illicit drugs is common among individuals with psychiatric problems [16, 42]. For this reason, it is important to find a valid and reliable tool for assessing alcohol use that can readily be used in mental health services [43]. The AUDIT is a 10-item self-report instrument designed to detect problem drinking [34] and is shown to be both reliable and valid among psychiatric patients [27, 30, 44–46].

The present study demonstrated that the prevalence of alcohol consumers among male and female Iranian psychiatric outpatients was 19 and 3.1%, respectively. The high proportion of hazardous consumers among them (above 60% for men and 30% for women) may alcohol is often used as self-medication among psychiatric patients [47]. Compared to the Iranian general population, the estimated prevalence of alcohol use was higher among men and lower among women [5], Risk drinking was estimated to be higher in both male and female patients. This is consistent with earlier surveys suggesting elevated rates of alcohol abuse among persons in treatment for mental illness [42]. These higher rates may be attributable to common dopamine neurotransmitter system that mediates both psychiatric and substance use disorders, or the role of some mental disorders in predisposing the development of substance abuse [47]. On the other hand, it may be assumed that people in medical settings have a greater tendency to report symptoms without inhibition because of concerns about well-being [27]. In a country like Iran where alcohol use is illegal and disrespected, it is likely that individuals will report consumption more accurately in therapeutic settings where perceived stigma may be lower compared to in the general population [27]. As alcohol consumption can drastically complicate treatment, more research is needed to determine accurate rates of AUDs in general populations and psychiatric samples in Iran.

The reliability (internal consistency) of AUDIT was high. Previous studies have examined reliability of both the original and non-English versions of the AUDIT and have consistently demonstrated acceptable reliability [37].

The Confirmatory Factor Analyses showed that the construct validity and the fit of the proposed factor structures (1, 2 and 3 factors) were not good and intuitively contradict the results of the exploratory principal axis factoring performed here. The exploratory factor analysis showed that a 1-factor solution explained 77% of the co-variance between AUDIT items. As the construct validity was ambiguous, the AUDIT unexpectedly seems not to have good psychometric properties in Persian psychiatric outpatients. One explanation may be the adaptation of the alcohol content (standard drinks) used in items 2 and 3 to Iranian conditions, making the item-total correlation pattern change. Another explanation may be the high inter-item correlations that were not permitted in the CFA model. A third explanation could be the very skewed distribution of alcohol consumption in the sample. AUDIT was constructed for use in populations with a lower proportion of lifetime abstainers. However, if AUDIT is used in this context as a single factor scale, the recommended cut-off for Hazardous drinking is 8+ in men and 6+ for women [41, 48]. The internal factor structure of the AUDIT that was suggested has presented three models (1, 2 and 3 factors). Most studies supported a two-factor model; one for consumption and another for problems and consequences of alcohol use [26, 38]. In a sample of psychiatric patients, using exploratory and confirmatory factor analysis, Carey et al. [30] recommended a one dimensional structure of the questionnaire. In samples with a high prevalence of alcohol dependence, most researchers suggest that AUDIT measures one construct (or two constructs in low prevalence samples) [26]. The result of our exploratory factor analysis resembled other samples showing a high prevalence of dependence, but was different from a sample that shows lower prevalence of dependence (should there be a reference here?), while the AUDIT appears to have a single-factor structure. One reason why we could not reproduce the suggested structure of AUDIT in Iranian psychiatric outpatients could also be that a small part of the patients drink alcohol all but among them a high proportion drinks hazardous.

### Limitations

This was the first attempt to compute the prevalence of alcohol use in a psychiatric outpatient population in Iran. As the sample was obtained from only one outpatient center, the generalizability of the results to other psychiatric patients should be considered with caution. AUDIT assesses self-reported alcohol habits, and alcohol use is illegal in Iran; thus, underreporting is likely, even among psychiatric patients. We did not evaluate clients’ alcohol use using alcohol biomarkers, which can increase the number of positively cases [49]. Another limitation is that we only examined a sample of patients, most of whom were life-time abstainers. The deletion of cases that the psychiatrist considered inconsistent would inflate the Cronbach alpha of the scale. The use of statistical methods appropriate for psychometric evaluations in a sample with a high proportion of lifetime abstainers may be discussed. More research is required to assess the validity and reliability of the AUDIT with psychiatric in/out patients with larger participant samples.

## Conclusions

The AUDIT exhibited good psychometric properties regarding internal reliability and when used as a single scale. This confirms the findings by Ghorbani et al. (2017) even if the alphas are somewhat inflated in the current study. The prevalence estimates were somewhat higher among psychiatric outpatients compared to the general population, which supports the construct validity. However, the dimensionality found in other drinking cultures could not be reached in an Iranian context. This may be due to the adoption of the ‘standard drinks’ used in other countries and the extremely skewed distribution of alcohol consumption with 80% lifetime abstainers, even among psychiatric patients.
